# Menstruation and period poverty as an obstacle for girls’ equal participation in education, Tanzania

**DOI:** 10.1186/s12889-025-24864-w

**Published:** 2025-10-28

**Authors:** Vibeke Vågenes, Cecilie Grevstad

**Affiliations:** 1 Faculty of Education, Arts and Sports, Western Norway University of Applied Sciences, Bergen, Norway; 2 Faculty of Education, University of Inland Norway, Hamar, Norway

**Keywords:** Menstrual Health Management, Period Poverty, School, Gender, Tanzania

## Abstract

Monthly menstruation can complicate participation and achievement for schoolgirls. On a global scale, and in Tanzania, school enrolment of girls and boys are becoming equal, or even in favour of girls. However, in contrast to many other countries, Tanzanian boys on average score better than girls on secondary school exams. We argue that menstrual health management (MHM) difficulties are probably a barrier to female participation and success in secondary education. Factors like cultural beliefs and taboos, poverty, inadequate infrastructure at school and at home, lack of pads, and of relevant knowledge, are challenging to girls who are pursuing an education and at the same time balancing norms and ideals of traditions and of modernity. We argue that knowledge and openness concerning menstrual health management is needed, and that the school has an essential part to play in this.

## Introduction

Menarche marks the embodied transformation from girlhood to womanhood. During the final years of primary school, and the formative years of secondary school, girls must learn to manage their bodily transformations. In Tanzania, the students spend long hours in school, which in most cases offers limited access to sanitary facilities that are basic to handle the monthly bleeding. In addition, buying safe menstrual products is beyond of the economic reach of many girls.

We are addressing the situation regarding menstrual health management (MHM) in schools in Tanzania, both from an individual and a structural perspective. We explore the extent to which handling menstruation is a barrier to personal development and freedom, and schooling of Tanzanian girls, something which ultimately relates to questions of justice and sustainability. Our research questions are:

1. How do secondary school girls in Tanzania handle and experience their menstruation?
2. In what ways does MHM represent a barrier for girls’ engagement with education?

There is a growing appreciation worldwide that girls’ equal participation in education requires that they have access to facilities where they can handle menstruation with dignity [1]. Access to toilets, water, soap and not the least, sanitary products such as pads, and undergarments is essential. When this is in place, at affordable costs, it is possible to realise sustainability goals and allow girls to reach their capabilities. However, MHM is also about whether the girls have access to empowering knowledge about their health.

Tanzania has invested much in achieving education for all and gender parity in education. The total national literacy rate was 76% in 2021, although there is a significantly lower literacy rate among women, compared to men [2]. In 2023 there was a net enrolment of 86% in primary schools [3]. The country has achieved gender parity in enrolment to primary school. During the recent decades the transition rates from primary to secondary school have been growing, although only around half of the students transfer to secondary school: in 2021 the gross enrolment rate of lower secondary education was 38.2%, whereas that of primary education was 98.7% (UNESCO Institute of Statistics). However, girls have a higher failure rate in the final exams of lower secondary education. This is reflected in the participation at the upper secondary and tertiary levels. In the last two years of secondary school (A-level/Forms 5 and 6), the majority (56%) of students are boys. A similar trend is evident in enrolment to university and other higher education [1, 2, 4, 5].

The gendered difference in academic performance in secondary schools in Tanzania is an important backdrop to this study. Except for Kiswahili, boys outperform girls in all subjects, and more girls than boys fail the school leaving exams [2]. Interestingly, the situation is the opposite in countries of the Global North, where girls clearly surpass boys. It is possible that girls’ MHM challenges contributes to this underperformance.

Tanzania is a poor country and despite years of economic growth, the reduction in poverty remains slow. The World Bank reported that almost half of the population lives under the poverty line. Generally, poor people have fewer years in school. The survey also reveals that 29,2% of the population have no access to ‘limited standard drinking water’, and that 71,5% have no access to ‘limited standard sanitation’ [6]. A report written by The Tanzanian National Institute for Medical Research (NIMR) [7] highlights that despite government efforts to waive value added tax on menstrual products, there is an absence of a specific policy or guidelines for menstrual health in the country. According to NIMR, school, water, sanitation and hygiene (SWASH) guidelines and school curriculum were the only policy tools in which MHM was a component.

The World Bank [1] calculates that on a global scale 500 million girls and women lack access to products and facilities that enable them to handle menstruation with dignity. In Tanzania, girls from poor households in general lack access to secure menstrual products and proper hygienic facilities [8–11],

### Conceptual clarification

Menstrual health management (MHM) relates to achieving several of the Sustainable Development Goals, particularly SDG4 (Ensure inclusive and equitable quality education and promote lifelong learning opportunities for all), SDG5 (Achieve gender equality and empower all women and girls) and SDG6 (Ensure availability and sustainable management of water and sanitation for all) [12].

The concept, ‘period poverty’, pertains to MHM of people living in poverty, or in situations where there is a lack of WASH facilities (water, sanitation, and hygiene facilities) and of sanitary products. American Medical Women’s Association defines period poverty as ‘the inadequate access to menstrual hygiene tools and educational materials, such as sanitary products, washing facilities, and waste management’ [11], 1). WHO/UNICEF defines MHM as:

Women and adolescent girls are using a clean menstrual management material to absorb or collect menstrual blood, that can be changed in privacy as often as necessary for the duration of a menstrual period, using soap and water for washing the body as required, and having access to safe and convenient facilities to dispose of used menstrual management materials. They understand the basic facts linked to the menstrual cycle and how to manage it with dignity and without discomfort or fear [13], 8).

Sacca et al. [11] suggest distinguishing between ‘hardware’ and ‘software’ period poverty. The former refers to material deprivation, such as lack of absorbents, or lack of water, soap, etc. The latter has to do with the accompanying lack of knowledge and education, and the psychosocial deprivation that many girls experience. Often this has to do with the sensitive nature of women’s menstruation, and the cultural taboo surrounding the matter. In our analysis we are distinguishing the material and the cultural contexts of MHM from the educational aspects, intending to clarify the task for the school in teaching girls and future mothers of daughters safe and dignified handling of their menstruation and reproductive health.

### Relevant research and theoretical perspectives on MHM

There is some useful previous research on how period poverty affects girls and women in East Africa [11, 14, 15] although more evidence is needed [16], especially concerning the lived experience of the girls. In the following we review relevant research according to the theoretical perspectives of material, knowledge and cultural context of MHM. These three categories derive from our analysis of data, and we will return to this. We argue that in all perspectives, MHM is placed in a tension between tradition and modernity.

### The material perspective

The average secondary school day in Tanzania starts around 7.30 am and may last until 4 or 5pm. In 2022, the schooldays were prolonged as an effort to achieve higher quality in education and ensure that students perform better at exams. Many students must walk long distances to and from school. In other words, menstruating girls need to rely on sanitary products that can keep them safe from bleeding through, or releasing odour, during long hours in school. They also need proper toilets or latrines, water, and soap, and preferably a private room where they can attend to their personal needs. If these elements are lacking, girls will be more likely to stay home during the menstrual period.

The situation is far from satisfactory, and most public schools do not meet the national norm for sanitary facilities. Statistically there are 52 pupils for every pit latrine, against the national standard of 1:25 [2]. Observations in schools confirm that school latrines often lack doors, privacy, and water. In urban schools, more than half of the schools lack basic WASH facilities, [8–10, 17, 18]. Only 44% of the schools have adequate toilets [19] and 54% of schools fail to provide water in connection with toilets. For the girl student, it is important that these facilities are in close vicinity to each other.

When it comes to the supply of sanitary products, the situation is difficult. The commercially available pads are mostly available in urban areas, and they are costly. A study from five countries in the region shows that commercial reusable pads are known only to a few girls and that most girls use available materials [20]. In Tanzania, they found that 84% of the surveyed girls use reusable materials (pieces of cloth, cotton, sponge). Some schools supply students with sanitary pads in emergencies. However, in the report from (NIMR [7], p. 53) it is shown that, although 41 per cent of schools provide free menstrual materials during emergencies, supplies are often inadequate and irregular due to funding shortages.

A study from peri-urban secondary schools in Uganda [21] shows that physical problems like pain and feeling unwell are among the most frequently reported reasons for girls staying at home during menstruation. ‘Sickness’ was the most common reason for girls’ absenteeism, while boys reported problems with paying school fees as the most common reason for staying home [21]. The study finds it difficult to quantify how many days girls stay at home due to menstrual problems, but their diary data concludes that there is a clear association between staying home from school and menstruation. Also, in their in-depth interviews, girls revealed that menstruation is an impediment to school attendance [21].

There is not sufficient evidence proving that girls are absent from schools or workplaces more frequently than their male counterparts, or that absenteeism is directly related to MHM, but several studies report that girls are staying at home some days every month [8–10, 17]. Distress caused by shame, anxiety, and stigma surrounding menstruation is strongly felt by the girls and may prevent them from going to school. A baseline study in five Sub-Saharan countries found that on average 50% of girls stay home during menstruation [20]. Miiro et al. [21] conclude that only 1,1% of the girls reported to have access to all necessary components (products, adequate disposal, water and soap) and do not feel anxiety about the next period. In addition, girls may be absent from school due to household chores and family obligations.

In our view, it is important to note that the MHM must be studied in the contexts of female living, because households’ lack of capital, lack of WASH-facilities and accessibility of products vary greatly between places, especially between rural and urban contexts. There is a need to document and understand if and how menstruation, and the gendered norms surrounding it, affect girls’ freedom to lead a dignified life. This issue relates to education, public health, and gender equity, and needs more research.

### The knowledge perspective

Hennegan et al. [22] conducted a systematic review of relevant literature, and found that most studies conclude that girls have knowledge deficits concerning menstruation, and seek practical knowledge concerning managing menses, pains, etc. Tamiru et al. [20] found that on average 66% of the girls surveyed (in five African countries) had not known of menstruation before it started. Similarly, Miiro et al. [21] reported that only 24% of girls had learned about the period before menarche. The start of menarche was therefore shocking and fearful to many. Most of the girls (80%) reported that their mothers were their primary source of information, followed by the school and media, respectively [20]. Adults may be ill-informed, or uncomfortable sharing information [18, 21, 23, 24] and some boarding schools have employed a matron, normally a woman in charge of domestic and health arrangements at the school. They are responsible for creating safe educational environments and providing students with information, especially on health-related issues. However, few schools have a matron, and their ability to properly care for the students may be limited. Otieno [25] emphasises that the diversity of challenges facing girls and young women, combined with a shortage of educational resources and facilities, places constraints on the ability of matrons to provide the ongoing care that girls need.

A trial study from Kenya found that provision of pads and sex- and health education had limited effect on school attendance [26]. However, they found that addressing menstrual health challenges will be more effective when it is part of comprehensive education programs addressing stigma and shame, access to products, inequitable gender norms, and sexual and reproductive health knowledge gaps collectively.

### The cultural perspective

A study from eastern Uganda, shows that schooling ‘is a form of cultural capital connoting a form of ‘modernity’ that children hope to convert into resources for a better life’ [8, 27]. Likewise, education is seen as valuable to children in Tanzania. For many girls, school is their best opportunity to achieve the life that they are dreaming of, although the employment insecurity is huge [28]. Sommer [14] found that the transition to being young women, which is brought about by the onset of puberty and menstruation, leads to a situation where girls find themselves between conflicting values relating to traditional practices and to modern gender roles which focus on freedom and educational attainment. This is a conflict between expectations to adhere to traditional norms, for example of respecting spatial confinement when menstruating, and modern society’s expectations of pursuing an education and career [14].

According to traditions and cultural practices, menstruation is not a subject for public discussion [20]. It is linked to ideas of femininity, fertility, and sexuality. Traditional ideas of fertility and purity of the unmarried girl are still strong in Tanzania, and research shows that notions of sexual readiness are associated with the onset of menstruation. Girls often hide the start of menstruation because they are afraid of being accused of sexual activity [22, 29]. Given the unequal power relations between girls and older boys or men, girls may be susceptible to engage in sexual relations that frequently involve financial support, often referred to as ‘sugar daddy’ relations.

Tamiru et al. [20] found that in the five countries which they studied, women are considered unclean during menstruation. In some of the cases (specifically in Tanzania) there are restrictions on what menstruating women can do. Handling animals or water sources, passing through planted fields, and participation in religious congregations, are among the culturally imposed taboos for menstruating women. Confinement of the menstruating woman is a common cultural trait. Blood or other bodily fluids or substances may be considered dangerous and a source of pollution [30]. Purity is a concern in most societies, and thus, blood may represent a threat to the social order of the world. Douglas shows how menstrual blood is considered harmful around the world. The natural consequence is confinement of the person who is shedding blood.

Belief in witchcraft is common in contemporary Tanzania [31] and menstrual blood can, according to such beliefs, be used to harm others. Menstruating women are vulnerable to witchcraft [32]. Against this background, scientific knowledge about bodily functions, including the physical process of menstruation, can be crucial for girls who want to complete school. The role of the school is fundamental in delivering such knowledge.

Meinert [27] explores the intersection between health, capital, and local perceptions of a good life in Uganda. What is considered healthy and, thus, reflecting high morals and a person’s ‘smartness’ are among other things expressions of cleanliness such as having a beautiful home, dressing well in clean, ironed clothes, shoes and smelling good. Beauty and cleanliness are prestigious forms of symbolic capital [27], and personal hygiene connects with this. Washing, bathing, and cutting fingernails symbolise high moral or symbolic capital. In school, as well as among neighbours, a person is valued according to personal appearance.

For a girl there is a lot at stake when handling bleeding in school. Handling MHM in a way that compromises the personal reputation may harm a girl’s body capital [27] and may have repercussions beyond the immediate shame. To appear as a learned person (schooled) requires demonstrating personal appearance in line with social expectations and cultural ideals. Cultural and symbolic capital is manifested in appearing as a learned person with a clean body and clothes, and with a nice smell.

For a girl it is also a matter of being ‘contained’ and in control. Mjaaland [33] describes how girls in Northern Ethiopia learn ‘to hold back’. As students in schools, they take care to avoid a dominant and outspoken role in the classroom, to speak with low voice, often not showing the teacher that they know the right answer to questions. Boys are encouraged to achieve and be competitive. A striking resemblance to Tanzania, concerns the lower academic performance of girls. Mjaaland argues that girls learn to ‘hold back’, to contain their capabilities and to not outperform boys. This way their bodily appearances are in line with ideals of femininity. Considering Butler’s [42] performative gender perspective, we focus on how girls and boys can ‘do’ gender differently, and that girls’ agency is hampered by norms and expectations [4], as well as material conditions.

## Methodology

This study draws on several sources of data. One of the researchers has 15 years of experience from Tanzanian educational institutions, engaging in research and practice placement of students. The materials from 2 master students’ projects are the essential elements [34, 35]. Data are sourced from three schools (private and public) from the same region and located in a rural and a semi-urban area. Girls (31), teachers (8), heads of schools (2), NGOs (3), other stakeholders (1), and representatives from local administrations (2), have been interviewed. In addition, the analysis includes data from 2 interviews with stakeholders associated with local non-governmental organisations and education administration, together with a range of informal interviews, field conversations and observations that we conducted through our teaching roles in the schools (participant observation). Conversations have been performed in English, and quoted excerpts from interviews are left unaltered.

Documents and statistics from national authorities have been analysed, including National Education and Training Policy [36], Basic Education Statistics [3], and University Education Statistics [5].

Our focus on girls and MHM has grown out of this long-term involvement in Tanzanian education. Understanding insiders’ views, and what Goffman [37] calls the backstage performance (revealing more of the true self), is time consuming requiring immersion in social contexts and trust between researcher and informants. MHM remains personal, sensitive, and in this case, staged in a culture where sexuality, health/reproductive health and bodily issues in general are not publicly discussed. We assume that ‘knowledge is embodied, engendered and embedded in the material context of place and space’ [38], or ‘situated’ [39]. Access to girls’ backstage experience of menstruation can be gained through observant participation and the embeddedness of the researcher [40]. All the involved researchers are female, and some are close to the girl students in age. Repeated periods of participation in the same institutions were important for building the necessary trust and relations that granted access to the situated knowledge of girls. The fieldwork period for the masters’ projects, a total of seven months, included student teachers’ practicum periods, as well as conventional fieldwork periods.

The data that make up the material for this study were collected in a manner approved by Norwegian Social Science Data Services (NSD) and consistent with the ethical guidelines of the National Committee for Research Ethics in the Social Sciences and the Humanities’ (NESH). Although this is social science research, the project is in line with the principles of the World Medical Association Declaration of Helsinki, respecting the health, dignity, integrity, autonomy, privacy, and confidentiality of the participants. The duty of confidentiality was guaranteed by the safe storage of notes and audio recordings. Most of the interviews were recorded, according to informed consent and ethical clearance. The names of participants, schools and institutions have been anonymised to prevent recognition, and some contextual information has been eliminated for the same reason.

The analyses of data can be described as qualitative and inductive. Software for analysing qualitative interview data was used for categorisation. Thematic categories were inductively developed in this process. In the following we will present and discuss data and results according to overarching thematic categories corresponding to those that were used in the review of relevant literature.

## Results and discussion

In her anthropological study, Meinert [27] highlights the transformative potential of education, as it relates to modernity and improved quality of life. Expected outcomes of education include a healthier population, and the emergence of children as change agents in their families [27]. It is the responsibility of the school to educate the children about health, how to prevent illness and live healthier lives. Social appearance and the construction of bodily presence is important. To appear as smart, clean, and tidy, is highly valued in Tanzania. By participating in schooling, children appropriate social practices associated with competent, educated, and modernised people, which in turn, is believed to enhance their opportunities in life. Employability is bolstered by proper appearance, and displaying good personal health is endowed with great cultural value. For instance, strict dress codes are enforced for public employees, teachers, and students. Accordingly, we explore how girls can participate in schooling, and how their cultural context, knowledge, and material circumstances intersect, allowing them to appear smart, modern, and dignified. We begin with an examination of their material conditions.

### Material situations

In field conversations, female stakeholders contended that menstruation contributes to lower school participation. Women recounted how they stayed home 3–5 days every month due to menstruation. Teachers in public schools also indicated that girls miss school a few days each month. Beatrice, a key informant, initiated a campaign and founded an NGO focused on improving MHM. She visits schools to talk about this. When asked whether she believed menstruation causes absenteeism, she highlighted culturally based perceptions of the female body and the associated feelings of shame.

Mhm, definitely! I would say here it’s a challenge. Actually, I wouldn´t, I would put like sickness like, aah, I would put it to maybe 20 percent [of the reason why girls stay home while bleeding] because of the stomach upset and the headaches and such. But the rest is because a girl feels ashamed [34], 60)^1^

One challenge that many girls face is the time they spend walking to and from school, adding to the many hours at school. Far from every local community has a secondary school. As a result, the walking distance to and from the school is often long. A teacher in a public secondary school argued:

A girl walking 7 to 8 kilometres, to and from school, and she is at menstrual period is a bit discouraging. So that's why most of them choose to stay at home during the menstrual period. [35], 51)

A Form 3 girl in a boarding school talked about her relatives in her rural home area without proper sanitary products available:

But it's not that easy, so you can´t find yourself. Sometimes it's three days. You're at home waiting for this menstruation period to be over. It's so many days you're at home. Yes, especially in those interior areas. Because someone, some people are not getting their sanitary pads, so they drop out from school and stay at home. [35], 52)

Interviews performed in the boarding school depicted how the girls have long days, without access to dormitory facilities between early morning and late afternoon.

Interviewer: So, what is it like having your period in school?

Student: Sometimes it is very difficult […] Ooh! An example is tomorrow you enter to class and then the dormitory has been closed until 2-2.40 pm. You are going to stay in class until that time, and you feel uncomfortable because we cannot go to change the pad. Even to stay in the class or to stand or to walk from…. You will stay in class like this [mimics standing still] [34], 43).

This student described that the dorms were locked from the beginning of the day (around 7.00 am) until the conclusion of classes. During these hours, the girls used common facilities. In this particular school the students had been provided with reusable pads; however, if they needed to change their pads, they had to find a way to store the used ones until they could return to the dormitory to wash them. This situation is problematic. However, most schools are not boarding schools, making the issue of menstrual hygiene management more pronounced in public schools. The lack of proper sanitary facilities has been described, and very often latrines are without water, and even doors, which compromise the privacy for the users.

Multidimensional poverty encompasses deprivation in three or more key dimensions, including nutrition, health, protection, education, information, sanitation, water, and housing [13], ix). UNICEF documented that 88% of Tanzanian children are deprived in at least three dimensions of well-being with more than 90% of the children living in inadequate housing and sanitation conditions. Poverty also affects public services and includes inadequate WASH facilities: for instance, many schools feature un-secluded latrines, lack of water and soap, or no proper facility to dispose of used pads.

For most girls, purchasing commercial sanitary products is financially unattainable. Many girls resort to using alternative methods, such as pieces of kanga cloth or, if fortunate, reusable pads. As mentioned previously, it is not uncommon that girls engage in sexual relations with the purpose of financing their material needs. For some this is with intention to buy sanitary products [15, 34, 35]. A small package of sanitary pads may cost 2500 Tanzanian Shillings, representing all, or most of a day’s earnings, or the equivalent of 1 kg of rice [34].

Poverty may force girls to resort to traditional methods that are less compatible with school. Here are the words of a Form 3 student when asked about problems of finding pads:

Yes, and some even just use a cloth because they cannot afford that pad. So, they just use some cloth. Because life is tough, some families don't have the economic. And sometimes it is just because of lack of parental care. Some parents do not care about their children, especially the girls. [35], 36).

Some NGOs have started to distribute packages of reusable pads in the region. One of these is run by MHM/reproductive health activist Beatrice, who, based on her personal experience, managed to start production of such pads in her own home. Below she talks about when she started menstruating and tried to stop the bleeding using local materials:

I saw a lot of blood and I was like ‘What is this? Maybe something is very wrong with me?’ Now what did I have to do? I thought of taking something like eeh… you know the maize cob? The cob after you remove the maize. I thought of inserting it to the ‘vivi’ [vagina] so that the blood would stop coming out. Hahaha. That I saw didn’t work. It was painful. Then I thought okay maybe I can use mattress or maybe the cow dung. I started using all of them. Then I got so infected. I got sick. I couldn’t tell my mum. There was no one I could have told because no one spoke about menstruation. Then I came up with an idea. A very clever one. I took the polythene bag, and then I put my brother’s t-shirt around, and then I put it down. And it was working […] So, it was very stressful. I thought of missing days to go to school. It was challenging. Until I got to secondary school, that is when I came to learn about menstruation.

Today, Beatrice creates MHM packages for distribution in schools as part of her educational program. These packages include two reusable pads, two pairs of underwear, soap, a washcloth, and a calendar to help track the menstrual cycle. As mentioned, she teaches schoolchildren about MHM. She always leaves time for questions from the children. Observations of her classroom activities revealed that the girls listened intently to Beatrice, indicating the relevance of the topic to their lives. Demonstrations of underwear caused especially much excitement among girls, suggesting that many girls are lacking proper underwear. Beatrice teaches girls how to wear and wash underwear and pads and takes as her starting point that there is a generational gap in knowledge when it comes to MHM She also emphasized that many mothers are not well-informed about MHM products.

Even though reusable pads are a sustainable and economical solution, they also need to be washed and dried, which can be difficult for many girls. A Form 5 student explained the problem:

It is very dangerous for us. Because you find a person in her menstruation and she has only two of those towels [reusable pads], but she has to wash one and dry them, but you found there is no sun. And we don’t have the ironing. So, they get like curled and at the end we got the UTI [Urinary tract infection] and fungus… [34], 39).

In fact, during numerous visits to the boarding school for girls, we have never seen a reusable pad that has been put out to dry. This is a known problem and relates to the cultural taboo of managing menstruation in public. The girls always put other clothes on top to dry their reusable pads. Extracts from one focus group discussion may explain the practice of covering the pads:

Girl 1: Because of fear.

All: Yeah because of fear.

Girl 1: Sometimes shy. “Eeh, if a man saw this, how would they…”.

Girl 2: Yeah, if a man saw this.

Interviewer: So, the problem is if a man sees it?

All: Yeah, yeah.

Interviewer: Mmm, even fathers and brothers also?

Girl 3: Yeah, yeah, haha. We respect them. [34], 56)

Students conceal the evidence of their menstruation as a sign of respect. In this hierarchical system, older men hold a position of authority, and demonstrating dignity and respect toward one's elders is an important aspect of the social order. There exists an acceptable standard of ‘proper menstrual behaviour’ within society, and girls learn to manage the shame associated with menstruating by concealing it to prevent causing discomfort to others.

### Body and knowledge

Social attitudes towards menstruation significantly impacts the information that girls receive, both at home and in school. Tanzanian girls typically obtain basic information about menstruation from their mothers or other female adults, friends, and schools. Even so, it is common that girls lack knowledge about menstruation before reaching the menarche [21]. This is a common experience of our informants. Although some girls received information from mothers or other female guardians, many reported feeling unprepared and uninformed upon reaching menarche. One girl in a focus group discussion noted, ‘Mom never told me anything, and I didn´t have a friend who have told me this and this, so I did not know anything. Yes, some parents are afraid to tell their children’ [35], 64). This lack of knowledge can lead to low self-esteem, anxiety, stress, or confusion, and even infections.

Beatrice is unusually outspoken concerning sexual and reproductive health issues. Through her NGO she is also running an education programme for local schools that presents knowledge about puberty, reproduction, and menstruation. She said that many families want her to inform their children about puberty, menstruation, and reproductive health. Furthermore, she explained that there are local myths concerning sex and menstruation. In her view, ‘every school has its gap of knowledge’, and teacher education does not really prepare student teachers to teach about reproductive health and body changes. Beatrice argues that there is a ‘culture of silence’. She also says that parents often avoid talking about topics of menstruation and sex because they never experienced their own parents talking about such issues.

In Tanzania, both the primary and the secondary school curricula include topics on reproductive health. Biology textbooks for Primary Grade 6, when students are 12–13 years of age, mention menstruation under ‘Maintaining health and environment’, including topics like ‘Applying principles for good hygiene, health and environment’ and ‘Identifying various systems in the human body’. Under this latter topic, the reproductive systems of boys and girls are dealt with in a factual manner. There is no mention of the practical, or emotional aspects of the having and managing the period. It is worth noticing that menstruation is connected to hygiene education, and that under the same topics there is mention of ‘The importance of cleanliness and smartness of garments’. In other words, the mention of menstruation goes together with ideas of cleanliness, hygiene and ‘smartness’. Cleanliness is generally an important ideal in the Tanzanian society and education. Many schools will send students home from school, or punish them, if they come to school in an improper or stained uniform. For some girls, adhering to these rules during their menstruation can be challenging, especially when they lack access to water or proper absorbent products.

In Secondary Form 3 textbooks, the issue of menstruation is addressed more directly. At this stage, the girls will be around 16–17 years old. The topic is included in the biology curriculum, specifically under the theme of reproduction, with a focus on the phases of the menstrual cycle. However, the textbooks do not provide practical guidance on menstruation; instead, they mention symptoms such as depression, stomach cramps, and irregular bleeding [34]. All interviewed girls agreed that Form 3 is too late to learn about menstruation. Some students have the option to take the elective subject *Nutrition* in Form 2, where the focus also is on the instrumental aspects and how to avoid pregnancy [35]. Here is an extract from one participant in a focus group discussion with girls in Form 3:

Yes, some girls get their menstruation period so early, even in primary schools. Because even for me, I didn´t know about menstruation up to Form 2. My first day when I got my menstruation, I did not know anything. I went to my mom and told her like, hey Mom, I think there is a difference with me I have seen some things that I don´t understand. [35], 65)

Menstruation being a neglected topic in education is not unique for Tanzania, but because of the culture of silence and the social attitudes that surrounds it, it is even more important for it to be introduced early in school, in an objective and informative way. We find that the silence that surrounds menstruation is ingrained in education and limits girls’ access to practical information. Sometimes, teachers who are consulted for advice may not have sufficient knowledge or be willing to guide the menstruating girl. Without statistical confirmation, our observations indicate that most science teachers are male, something which seems to complicate the flow of information.

Considering the lack of knowledge, the cultural taboo surrounding menstruation, as well as the folk beliefs concerning bodily fluids, a headmaster in a secondary school argued that there is 'an educational challenge' to meet what he calls superstition. He declared that students need to be educated because traditional beliefs and modern knowledge collide in the girls' handling of menstruation, inflicting fear that if they spill blood 'they will never stop bleeding again' [35], 65).

Witchcraft belief is a concern for many, and one of the girls participating in a focus group discussion explained the fear to use disposable pads:

Some do not like to use the disposal ones because they believe some superstition that if they throw away the ones that are used the witch will come and take their blood and do something, so they believe in superstition. [35], 63)

Witchcraft is a significant factor in folk beliefs, although strongly opposed in school. The lack of scientific knowledge is a major issue to the activist Beatrice in her campaigns. She was trained as a teacher, and her concern is for children to learn about what is happening to their bodies in an open and informed way. She is also concerned that what she sees as myths and misconceptions, for example witchcraft, should be replaced through non-judgemental dialogue in the school.

To summarise so far, widespread financial constraints, together with knowledge deficits, and social norms, complicate the management of menstruation for schoolgirls. In addition, there are also cultural taboos that need to be studied.

### Managing the body and cultural taboos

As mentioned, cultural beliefs suggest that shedding blood renders a person vulnerable to witchcraft. The girls who participated in the interviews concurred that when using non-reusable pads, the material that contains blood must be burned, otherwise witches may inflict harm to you if they get hold of your blood. One girl referred to one example saying that ‘you may lack children in the future’. Another said ‘You know the witch-people? They use that blood to… your blood is more valuable than everything…’ [34], 59).

On the other hand, the girls agreed that menstruation should not stop them from going to school. Nevertheless, they argued that it does influence their performance and participation, how they are ‘doing gender’ in school. They expressed fear to stand up in class, something that is usual when answering questions from teachers. One girl in Form 3 explained how she is anxious to answer questions and stand up in class:

To me I think ‘Aah, I am standing… Haha, if I stand I must…’ [stands up to show her checking her skirt]. And if I check myself Madam will know that I am looking, so even if I know the question, I do not want to raise my hand because I know Madam will… Madam will choose me, and I am afraid for Madam to choose me. Especially if there are male teacher, I am afraid to raise up my hand. I am just staying, and if I know the question, I can even tell my neighbour to tell instead of me [34], 50).

It is not only the fear of blood stains or odours from leaks that compels girls to restrain their physical appearance in school. Classroom observations frequently reveal that girls are often shy and speak in very low voices. Regional examples illustrate this point; Mjaaland (33, p. 87) describes how Ethiopian girls learn to "hold back" and "keep quiet" in the classroom, linking this behaviour to ideals of femininity associated with virginity, modesty, and honour. As girls reach puberty, they are often viewed as being at the height of attractiveness or desirability. Control of body posture and physical appearance is of outmost significance, also in Tanzania. Proper female behaviour extends also to menstrual behaviour. Girls learn shame and to conceal bodily conditions, pain, and problems that could otherwise indicate female reproductive, or sexual ‘readiness’.

## Conclusions

Our findings indicate that absence from school because of MHM challenges may negatively impacts girls’ educational careers and academic results. HakiElimu’s Annual Report [41] points out that girls need reusable sanitary pads and proper WASH-facilities in school to ensure that they remain in school. Period poverty refers to the lack of reusable or disposable pads among schoolgirls. Many girls find it difficult to stay for long days in school without the possibility to change pads, wash and handle the materials in privacy. For such reasons many girls stay home several days every month. When they are not in class, they fall behind in the curriculum. We argue that there is a high probability that period poverty contributes to girls’ lower academic performance and their lower participation in upper secondary and tertiary education. One explanatory factor may be that girls reach the menarche in a situation of period poverty and with a lack of sanitary facilities in school. This could be explored further through survey research.

To understand period poverty, it is essential to grasp the implications of the material situation of MHM. Some NGO programmes assist by providing girls with tools for menstrual management. National efforts to improve WASH facilities in schools also significantly support dignified MHM and can contribute to alleviating the spatial confinement experienced by menstruating girls. A stronger connection between education and health at the policy level could further facilitate this development.

Our research also shows that the cultural and social context in which girls grow up is important for their experience of MHM. For many girls in Tanzania, transitioning to womanhood involves restrictions on spatial movements due to expectations of adhering to traditional norms. Fear, shame, and taboos impact girls´ menstrual experience. We show how the social construction of ‘smartness’ and becoming a modernised, employable person through education, contrasts with traditional ideas.

Considering the cultural context, it is important to address the knowledge gap and the need for more comprehensive MHM education. Apart from the material aspect or ‘hardware’ period poverty, it also refers to the knowledge deficit, lack of education or support that many girls experience. Our findings suggest that many girls reach menarche with insufficient knowledge and support. Because of the culture of silence that surrounds menstruation, schools and teachers have an essential role in delivering scientific knowledge and creating supportive environments.

Summarising our research questions, we argue that girls in Tanzania experience that managing menstruation can be difficult in their material, social and cultural contexts. We also contend that education comes with an additional cost for girls, and that the monthly period should be seen as one of many barriers that girls face when seeking educational advancement, as they navigate the tensions between tradition and modernity.

## Data Availability

The data materials are not publicised, or made available for anybody, but the researchers. Interview data were recorded, transcribed and subsequently the recordings were deleted. All data are anonymised.

## Footnotes

1 Citation from interviews and focus group discussions have not been grammatically edited, and appears in direct transcription.
